# The role of Nrf2 in anoikis resistance and metastasis in anaplastic thyroid carcinoma

**DOI:** 10.1186/s43556-025-00355-7

**Published:** 2025-11-10

**Authors:** Simiao Fan, Huangcan Li, Ke Li, Zhongqin Gong, Xianhai Zeng, Shan-shan Wang, Yang Li, Chunlei Nie, Charles Andrew van Hasselt, Jason YK Chan, Michael Chi Fai Tong, George Gong Chen

**Affiliations:** 1 Department of Otorhinolaryngology, Head and Neck Surgery, The Chinese University of Hong Kong, Prince of Wales Hospital, Hong Kong SAR, China; 2 Shenzhen Key Laboratory of ENT, Institute of ENT & Longgang, ENT Hospital, Shenzhen, China; 3 School of Pharmaceutical Sciences, Guangzhou Medical University, Guangzhou, China; 4 School of Life Sciences and Biopharmaceutics, Guangdong Pharmaceutical University, Guangzhou, China; 5 The Shenzhen Research Institute (SZRI), The Chinese University of Hong Kong, Shenzhen, China; 6 The Third Affiliated Hospital of Harbin Medical University, Harbin, China

**Keywords:** Anaplastic thyroid carcinoma (ATC), Nrf2, Anoikis resistance, BCL-2, SLC7A11, Therapeutic target

## Abstract

Anaplastic thyroid carcinoma (ATC) is one of the most aggressive endocrine malignancies, characterized by rapid progression, extensive metastasis, and extremely poor prognosis. Despite advances in molecular oncology, the mechanisms driving ATC metastasis and therapeutic resistance remain largely unclear. Cancer cells that detach from the extracellular matrix must evade a specific form of apoptosis known as anoikis, and the ability to survive under these anchorage-independent conditions is a critical prerequisite for metastatic dissemination. The transcription factor nuclear factor erythroid 2-related factor 2 (Nrf2) has emerged as a master regulator of oxidative-stress responses and tumor adaptation, yet its function in governing anoikis resistance in ATC is not well understood. Here we demonstrate that Nrf2 expression is markedly upregulated in ATC tissues and cell lines, where its nuclear translocation drives transcriptional activation of anti-apoptotic and redox-protective genes including *BCL-2 and SLC7A11*. Under detachment stress, Nrf2 activation enhances cell viability, inhibits apoptosis, and facilitates multicellular aggregate formation, thereby promoting survival. Conversely, genetic silencing or pharmacological inhibition of Nrf2 with Brusatol markedly suppresses proliferation, invasion, and *in vivo* liver metastasis. Collectively, these findings identify Nrf2 as a pivotal driver of ATC anoikis resistance and metastatic competence through regulation of the BCL-2/SLC7A11 axis. Targeting the Nrf2-dependent survival pathway may thus offer a promising therapeutic strategy for this otherwise refractory malignancy.

**Supplementary Information:**

The online version contains supplementary material available at 10.1186/s43556-025-00355-7.

## Introduction

Anaplastic thyroid carcinoma (ATC) is a rare yet highly aggressive form of thyroid cancer, characterized by undifferentiated cells with stem-like features. Although accounting for less than 5% of all thyroid malignancies, ATC is the most lethal subtype, largely due to the lack of established treatment strategies and standardized protocols [1]. According to Surveillance, Epidemiology, and End Results data [2], the five-year overall survival for ATC patients is only 8%, with a median survival of just 3–7 months [3]. No current therapies significantly prolong overall survival, and over 40% of patients present with distant metastases at diagnosis [3], with common sites including the lungs, liver, and bones [4]. Mortality often results from local airway obstruction or metastatic complications [5], and management primarily relies on palliative interventions or site-directed targeted therapies [6].

Nuclear factor erythroid 2–related factor 2 (Nrf2) is a transcription factor that regulates antioxidant response elements (AREs) and maintains cellular redox homeostasis. Its dysregulation has been increasingly implicated in cancer progression [7]. While Nrf2 supports normal cell survival under stress, its persistent activation in cancer is linked to malignant progression, metastatic potential, and chemotherapy resistance [8]. Elevated Nrf2 activity is associated with aggressive behavior in multiple cancer types, including lung [9], breast [10], and cervical cancers [11], highlighting its dual role as both cytoprotective and oncogenic. In thyroid cancers, increased Nrf2 expression correlates with enhanced proliferation, invasiveness, and poor prognosis [12]. Furthermore, Nrf2 is recognized as a key regulator of autophagy, modulating autophagy-related gene expression to help cancer cells adapt to metabolic and oxidative stress [13], and has been proposed as a diagnostic biomarker and predictor of lymph node metastasis [14]. In ATC, preliminary studies have shown that Nrf2 silencing impairs cell viability, migration, invasion, and tumorigenic potential [15]; however, the precise molecular mechanisms by which Nrf2 modulates ATC progression and metastasis remain poorly defined.

Anoikis, a specialized form of apoptosis triggered by detachment from the extracellular matrix, serves as a critical barrier against metastasis [16]. Resistance to anoikis enables cancer cells to survive detachment, circulate through the bloodstream or lymphatic system, and establish metastases at distant sites [17]. Growing evidence indicates that crosstalk among redox signaling, metabolic adaptation, and cell death pathways, including autophagy and ferroptosis, contributes to anoikis resistance in cancer [18, 19]. Nevertheless, how transcriptional regulators such as Nrf2 coordinate these survival programs to confer anoikis resistance in ATC remains largely unexplored.

Building on our previous findings that Nrf2 regulates ATC cell viability and clonogenicity [15], we now demonstrate that Nrf2 knockdown impairs migration, invasion, and *in vivo* tumorigenesis. Using RNA sequencing and patient tissue analysis, we show that Nrf2 expression positively correlates with anoikis resistance–associated genes in ATC. Mechanistically, Nrf2 is activated under anoikis-inducing conditions, translocates to the nucleus, and directly upregulates BCL-2 and SLC7A11, thereby promoting anoikis resistance. Functionally, Nrf2-driven anoikis resistance enhances ATC cell migration and proliferation *in vitro* and supports metastasis *in vivo*, whereas its inhibition suppresses these malignant traits. Collectively, our findings reveal a previously unrecognized role for Nrf2 in anoikis resistance and underscore its potential as a therapeutic target in metastatic ATC.

## Results

### High Nrf2 expression in ATCs is associated with anoikis resistance

**Fig. 1:**
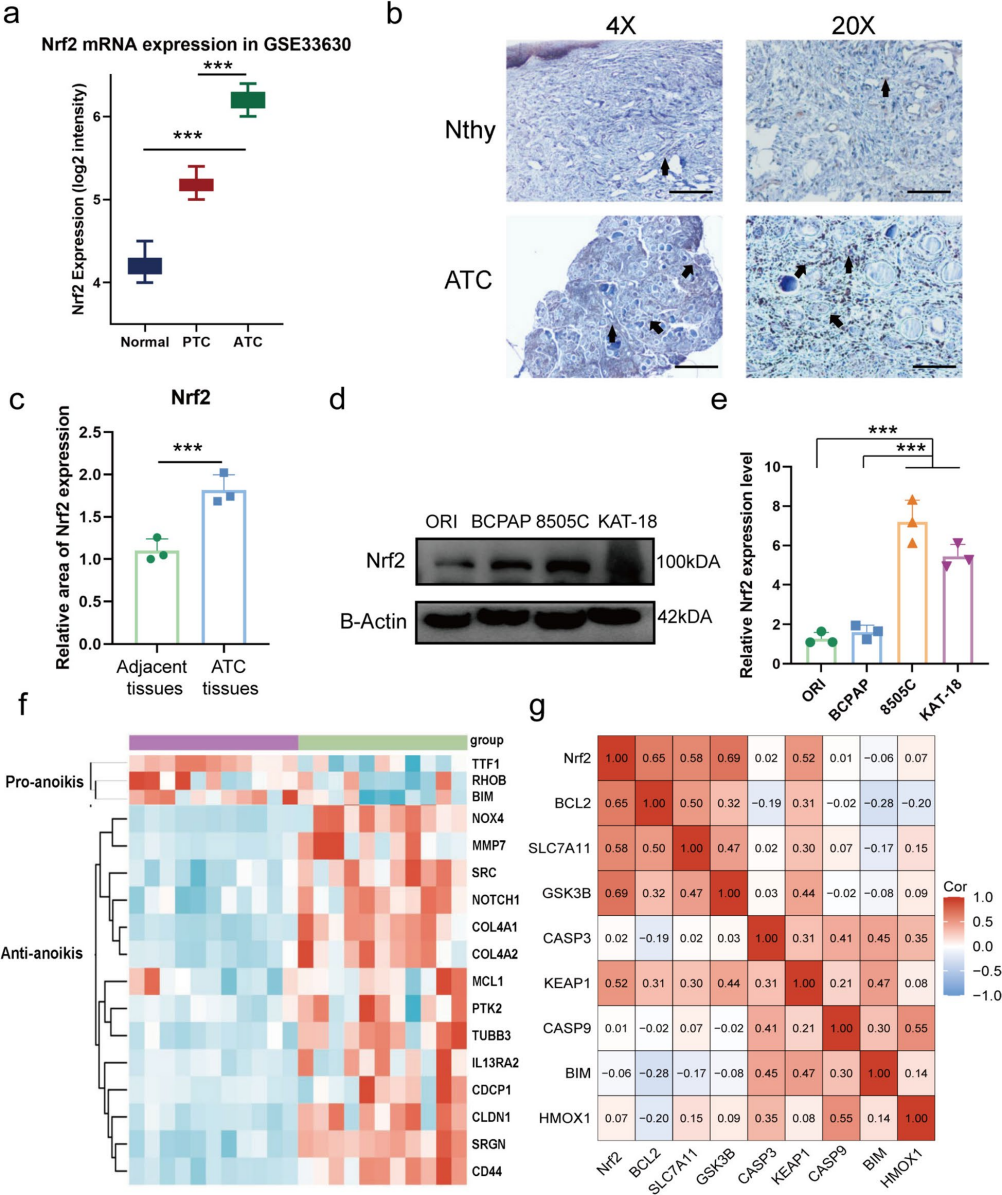
High Nrf2 expression in ATCs is associated with anoikis resistance. **a** Nrf2 mRNA expression in normal thyroid tissues (*n* = 45), papillary thyroid carcinoma (PTC, *n* = 49), and anaplastic thyroid carcinoma (ATC, *n* = 11) from the GSE33630 dataset. **b**-**c** Immunohistochemistry (IHC) of Nrf2 in ATC tissues (*n* = 6) and paired adjacent non-cancer tissues (*n* = 3), shown at 4×(Scale Bar, 100 µm) and 20×(Scale Bar, 20 µm). Arrows mark Nrf2-positive cells. **d**-**e** WB of Nrf2 in ATC (8505C, KAT-18), PTC (BCPAP), and normal thyroid (Nthy-ori 3-1) cell lines. **f** Correlation between pro-anoikis and anti-anoikis markers in ATC patients. **g** Correlation between Nrf2 and apoptosis-related genes. Compared with the corresponding control group, ***P < 0.001

To validate the upregulation of Nrf2 in ATC [15], we analyzed the GSE33630 dataset [20]. Nrf2 mRNA levels were significantly higher in ATC samples compared to both PTC and normal thyroid tissues (Fig. 1a). Consistently, immunohistochemistry (IHC) and western blot analysis confirmed increased Nrf2 protein expression in ATC patient tissues and cell lines (KAT-18, 8505 C) relative to adjacent non-cancerous tissues, normal thyroid cells, and PTC cells (Fig. 1b-e).

Given the established role of anoikis resistance in metastasis [17], and prior links between Nrf2 and anchorage-independent growth in other cancers [21, 22], we investigated its potential role in ATC. Analysis of the GSE33630 dataset revealed a distinct anoikis-related gene signature in ATC: pro-anoikis markers (TTF1, BIM, RHOB) were downregulated, while anti-anoikis markers (NOX4, MMP7, SRC) were upregulated (Fig. 1f). Furthermore, Nrf2 expression positively correlated with the anti-anoikis marker BCL-2 and its regulator KEAP1 and negatively correlated with the pro-anoikis factor BIM (Fig. 1g). These findings strongly suggest an association between Nrf2 and anoikis resistance in ATC.

### Nrf2 is induced under anoikis conditions and protects ATC cells from apoptosis

**Fig. 2:**
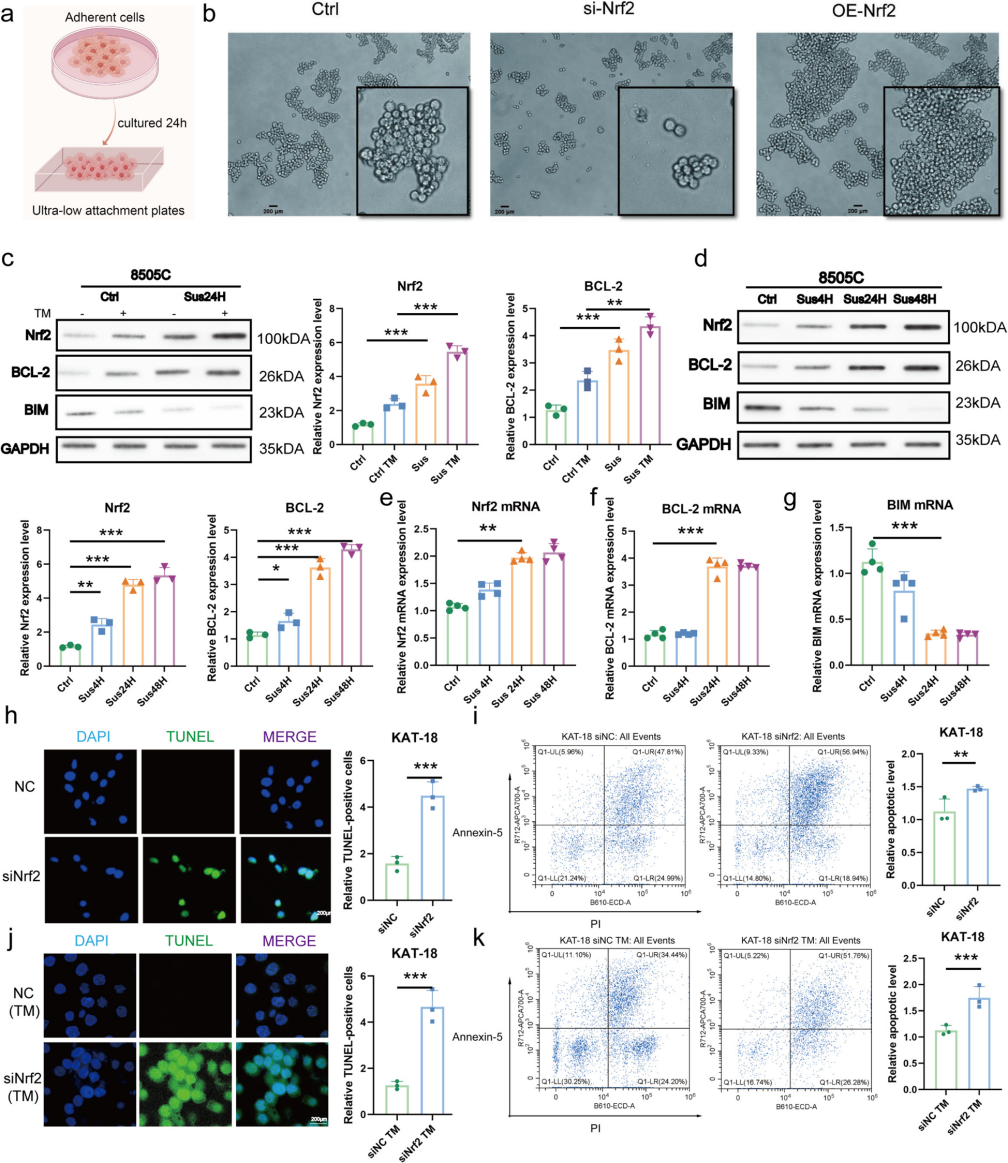
Nrf2 is induced under anoikis conditions and protects ATC cells from apoptosis. **a** Schematic illustration of the anoikis model construction. **b** ATC cells cultured in suspension were imaged at the indicated time points using a Zeiss inverted microscope. Scale Bar, 200 µm. **c** WB analysis of apoptosis-related proteins in ATC cells suspended for 24 h after TM induction. Protein levels of Nrf2 and BCL-2 were quantified by densitometry and normalized to GAPDH. **d** WB analysis of apoptosis-related proteins in ATC cells suspended for 4 h, 24 h, and 48 h. Protein levels of Nrf2 and BCL-2 were quantified by densitometry and normalized to GAPDH. **e**–**g** RT-qPCR analysis of Nrf2 and apoptosis-related mRNA expression in ATC cells suspended for 4 h, 24 h, and 48 h. **h** TUNEL staining showing increased DNA fragmentation in KAT-18 cells following Nrf2 knockdown, with corresponding quantification shown to the right. Scale bar, 200 µm. **i** Flow cytometry analysis of apoptosis under the same conditions, with corresponding quantification shown to the right. **j**–**k** Apoptosis analysis following Nrf2 knockdown combined with TM stimulation, including TUNEL staining, quantification (**j**), flow cytometry and quantification (**k**). The values are the means ± standard error of at least three separate studies. Compared with the corresponding control group, *p < 0.05, **P < 0.01, ***P < 0.001

To functionally assess the role of Nrf2 in anoikis, we utilized Tetrathiomolybdate (TM), a compound reported to promote anoikis [23]. Under TM-induced stress, Nrf2 knockdown sensitized ATC cells to anoikis, whereas Nrf2 overexpression significantly enhanced resistance, evidenced by an 82% increase in IC₅₀ (Fig. S1a-b). We next modeled detachment by culturing cells in suspension (Fig. 2a). Nrf2 silencing disrupted spheroid integrity, producing smaller and more dispersed clusters, whereas its overexpression promoted the formation of compact aggregates (Fig. 2b). At the molecular level, suspension culture induced a time-dependent upregulation of Nrf2 protein, which was further enhanced by TM treatment (Fig. 2c-d). This increase in Nrf2 was accompanied by a corresponding elevation of the anti-apoptotic protein BCL-2 and suppression of the pro-apoptotic protein BIM. Consistent trends were observed at the mRNA level (Fig. 2e-g). To directly quantify apoptosis, TUNEL assays and flow cytometry were performed. Nrf2 knockdown significantly increased the rate of apoptosis (Fig. 2h-i), an effect that was potentiated by TM treatment (Fig. 2j-k). Collectively, these data demonstrate that Nrf2 is induced by anoikis stimuli and is essential for ATC cell survival under anoikis stress.

### Nrf2 promotes anoikis resistance through KEAP1 dissociation, nuclear translocation, and ARE activation

**Fig. 3:**
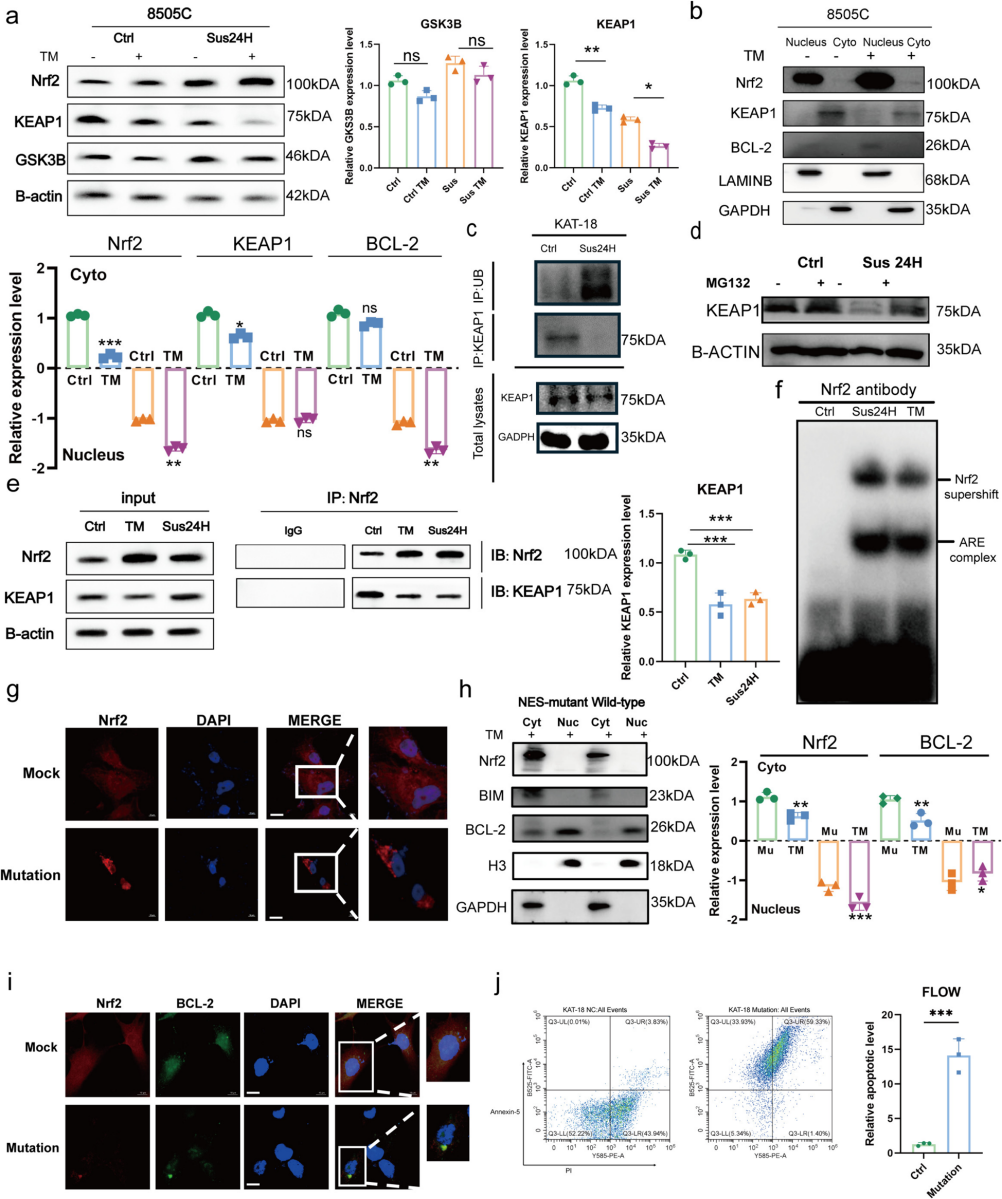
Nrf2 promotes anoikis resistance through KEAP1 dissociation, nuclear translocation, and ARE activation. **a** WB analysis and quantification of GSK3B and KEAP1 in ATC cells under the indicated conditions. **b** Nuclear and cytoplasmic fractionation blots and quantification showing the distribution of Nrf2, KEAP1, and BCL-2 in ATC cells. **c** Ubiquitination of KEAP1 detected by immunoblotting anti-KEAP1 immunoprecipitates with anti-ubiquitin antibodies in KAT-18 cells suspended for 24 h. **d** WB analysis of KEAP1 protein levels in the presence or absence of MG132 after suspended for 24 h. **e** Co-immunoprecipitation and quantification of the Nrf2-KEAP1 interaction under TM stimulation and suspended for 24 h. **f** Supershift EMSA analysis demonstrating Nrf2-specific ARE binding activity. **g** Immunofluorescence (IF) analysis showing that wild-type (WT) Nrf2 localized to the nucleus, whereas the NES-mutant Nrf2 remained cytoplasmic, confirming loss of nuclear translocation capability. Scale bar, 20 µm. **h** WB analysis and quantification of Nrf2 and BCL-2 in ATC cells transfected with WT or mutant Nrf2 plasmids under TM treatment. **i**-**j** Immunofluorescence analysis of BCL-2 TUNEL assay, and quantification of apoptosis in ATC cells after transfection with WT or mutant Nrf2 plasmids. Scale Bar, 20 µm. The values are the means ± standard error of at least three separate studies. Compared with the corresponding control group, *p < 0.05, **p < 0.01, ***p < 0.001, ns: not significant

We first determined that the canonical oxidative stress pathway was not responsible for Nrf2 activation under anoikis, as ROS levels were monitored in ATC cells under suspension conditions at multiple time points (0.5–48 h) using DCFH-DA and flow cytometry (Fig. S1c-d). No significant changes were detected compared with adherent controls, while H₂O₂ induced a clear increase as a positive control. Furthermore, the antioxidant NAC did not impair Nrf2 nuclear translocation (Fig. S1e). These data indicate that ROS is not a major upstream driver of the observed phenotype. We therefore investigated alternative mechanisms. Under anoikis-inducing conditions (TM + suspension), Nrf2 protein levels increased while KEAP1 decreased; GSK-3β levels were unaffected (Fig. 3a), suggesting KEAP1-mediated regulation.

Nuclear-cytoplasmic fractionation confirmed that TM stimulation promoted Nrf2 nuclear accumulation, concomitant with a reduction of cytoplasmic KEAP1 and Nrf2. BCL-2 levels also increased in the nuclear fraction (Fig. 3b). We further found that suspension culture enhanced KEAP1 ubiquitination (Fig. 3c) without altering its mRNA (Fig. S1f). Given that KEAP1 degradation is mediated by the proteasome [24], proteasomal inhibition by MG132 rescued KEAP1 protein levels (Fig. 3d), confirming proteasomal degradation without changing its mRNA (Fig. S1g). Co-immunoprecipitation revealed a marked reduction in Nrf2-KEAP1 interaction upon TM stimulation (Fig. 3e), consistent with ubiquitination-induced complex dissociation prior to degradation. Although the input lane showed only modest changes in total KEAP1 at this early time point, the binding between Nrf2 and KEAP1 was markedly reduced. This is consistent with a temporal sequence in which ubiquitination and conformational changes first weaken KEAP1–Nrf2 interaction, followed by compartment-specific KEAP1 degradation that is more apparent in cytoplasmic fractions and under MG132 rescue conditions (Fig. 3d). Upon oxidative stress, Nrf2 dissociates from KEAP1, translocates to the nucleus, and activates transcription via antioxidant response elements (AREs) [25]. To further verify the regulation of Nrf2 within the nucleus, EMSA confirmed enhanced binding of Nrf2 to AREs following TM stimulation and suspension culture (Fig. 3f). To verify that Nrf2 mediates anoikis resistance via nuclear translocation, cells were transfected with an NES-deleted Nrf2 mutant and treated with TM. This mutant retains Nrf2 in the cytoplasm (Fig. 3g), and its expression leads to a significant upregulation of the pro-apoptotic protein BIM and increased apoptosis, despite varying effects on BCL-2 protein levels (Fig. 3h-j). These results indicate that Nrf2 nuclear translocation is required for its transcriptional suppression of apoptosis and promotion of anoikis resistance.

### Nrf2 promotes anoikis resistance mainly through BCL-2–mediated anti-apoptotic signaling and partly via SLC7A11

**Fig. 4:**
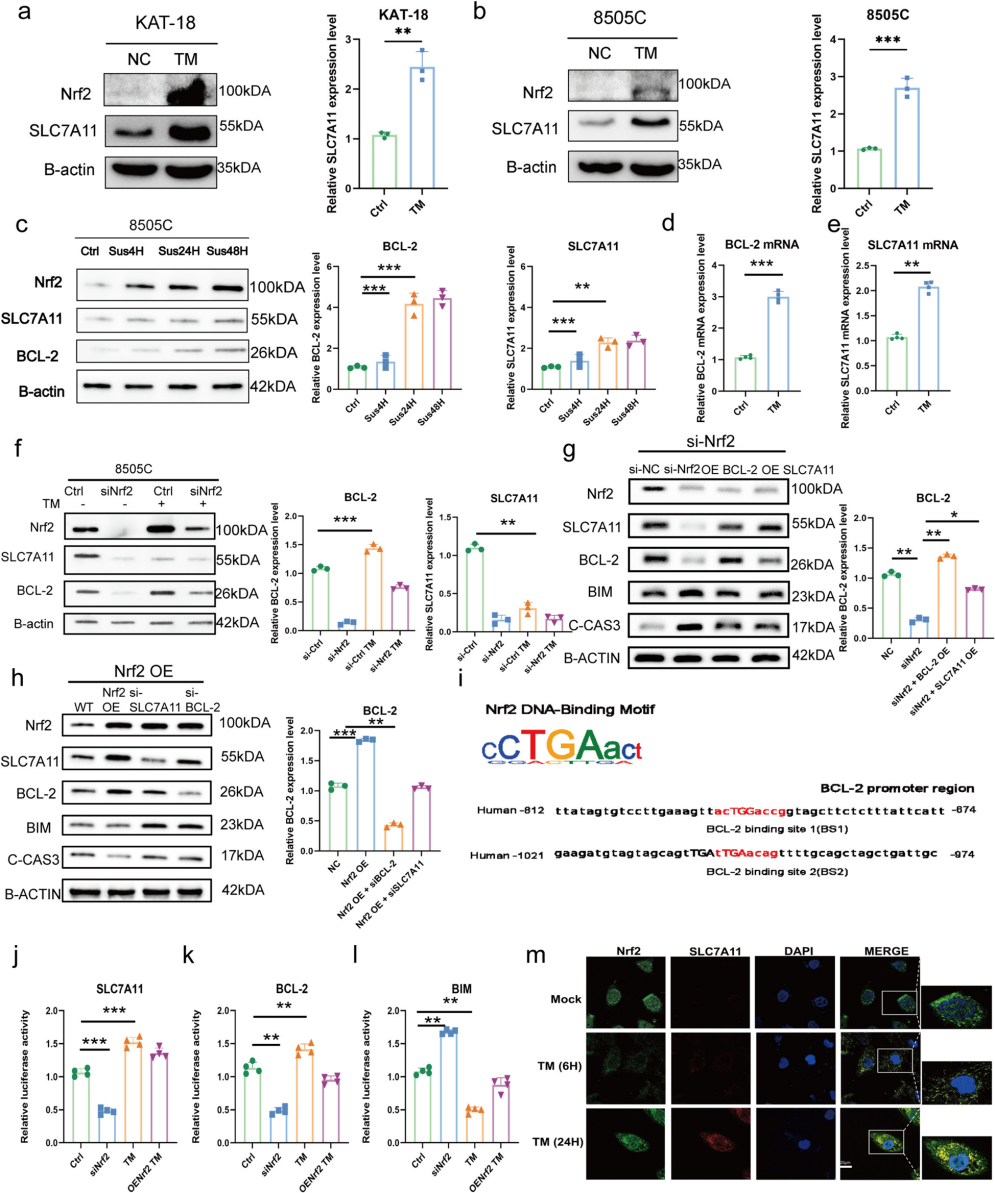
Nrf2 promotes anoikis resistance mainly through BCL-2–mediated anti-apoptotic signaling and partly via SLC7A11. **a**-**b** WB analysis and quantification of SLC7A11 expression in KAT-18 and 8505 C cells after TM induction. **c** WB analysis and quantification of BCL-2 and SLC7A11 expression in ATC cells suspended for 4 h, 24 h and 48 h. **d**-**e** The mRNA levels of BCL-2 and SLC7A11, when TM induced, were analyzed by RT-qPCR (*n* = 4 independent experiments). **f** WB analysis and quantification of BCL-2 and SLC7A11 in control and Nrf2-knockdown ATC cells with or without TM treatment. **g** WB analysis of anoikis-related markers in 8505 C cells transfected with siNC, siNrf2, siNrf2 + BCL-2 overexpression, or siNrf2 + SLC7A11 overexpression, with corresponding quantification shown to the right. **h** WB analysis of anoikis-related markers in 8505 C cells transfected with NC, Nrf2 overexpression, Nrf2 overexpression + siBCL-2, or Nrf2 overexpression + siSLC7A11, with corresponding quantification shown to the right. **i** Putative ARE sites in BCL-2 promoter were predicted using JASPAR database. j**-**l Luciferase reporter assays were performed using independent promoter constructs of SLC7A11, BCL-2, and BIM to evaluate transcriptional activity following Nrf2 knockdown and/or TM treatment in ATC cells. **m** The co-localization of Nrf2 and SLC7A11 after TM induction was analyzed by immunofluorescence. Scale Bar, 20 µm. The values are the means ± standard error of at least three separate studies.Compared with the corresponding control group, * *P*<0.05, ** *P*<0.01, ***p < 0.001

We next explored downstream effectors mediating Nrf2-driven anoikis resistance. Given the established role of SLC7A11 in maintaining redox balance during detachment stress [26], its expression was examined together with BCL-2. TM treatment markedly increased Nrf2 and SLC7A11 protein levels in ATC cells (Fig. 4a-b). Time-course analyses revealed that BCL-2 was rapidly upregulated after anoikis induction, whereas SLC7A11 increased more gradually (Fig. 4c). Consistently, qPCR showed stronger transcriptional induction of *BCL-2* than *SLC7A11* (Fig. 4d-e). Silencing Nrf2 reduced the basal expression of both genes and attenuated their upregulation under TM stimulation (Fig. 4f). Functional rescue experiments demonstrated that the expression of either BCL-2 or SLC7A11 in Nrf2-silenced cells partially restored protein levels and suppressed the pro-apoptotic markers BIM and cleaved-caspase-3 (Fig. 4g). Conversely, knockdown of either target in Nrf2-overexpressing cells re-sensitized them to apoptosis (Fig. 4h).

To determine whether Nrf2 directly regulates these genes, promoter analyses and luciferase assays were performed. Putative AREs were identified in the *BCL-2* promoter (Fig. 4i). Nrf2 activation enhanced, whereas its knockdown reduced, *BCL-2* and *SLC7A11* promoter activities (Fig. 4j-l), confirming transcriptional regulation. Direct Nrf2 binding to the promoters of BCL-2 and SLC7A11 has been demonstrated by chromatin immunoprecipitation coupled with quantitative PCR (ChIP-qPCR) and chromatin immunoprecipitation sequencing (ChIP-seq) analyses in previous studies [27–29]. Finally, immunofluorescence showed nuclear accumulation of Nrf2 and its co-localization with SLC7A11 under suspension conditions (Fig. 4m), further supporting their functional link in the anoikis response.

### Silencing Nrf2 suppresses ATC growth and metastasis* in vitro.*

**Fig. 5:**
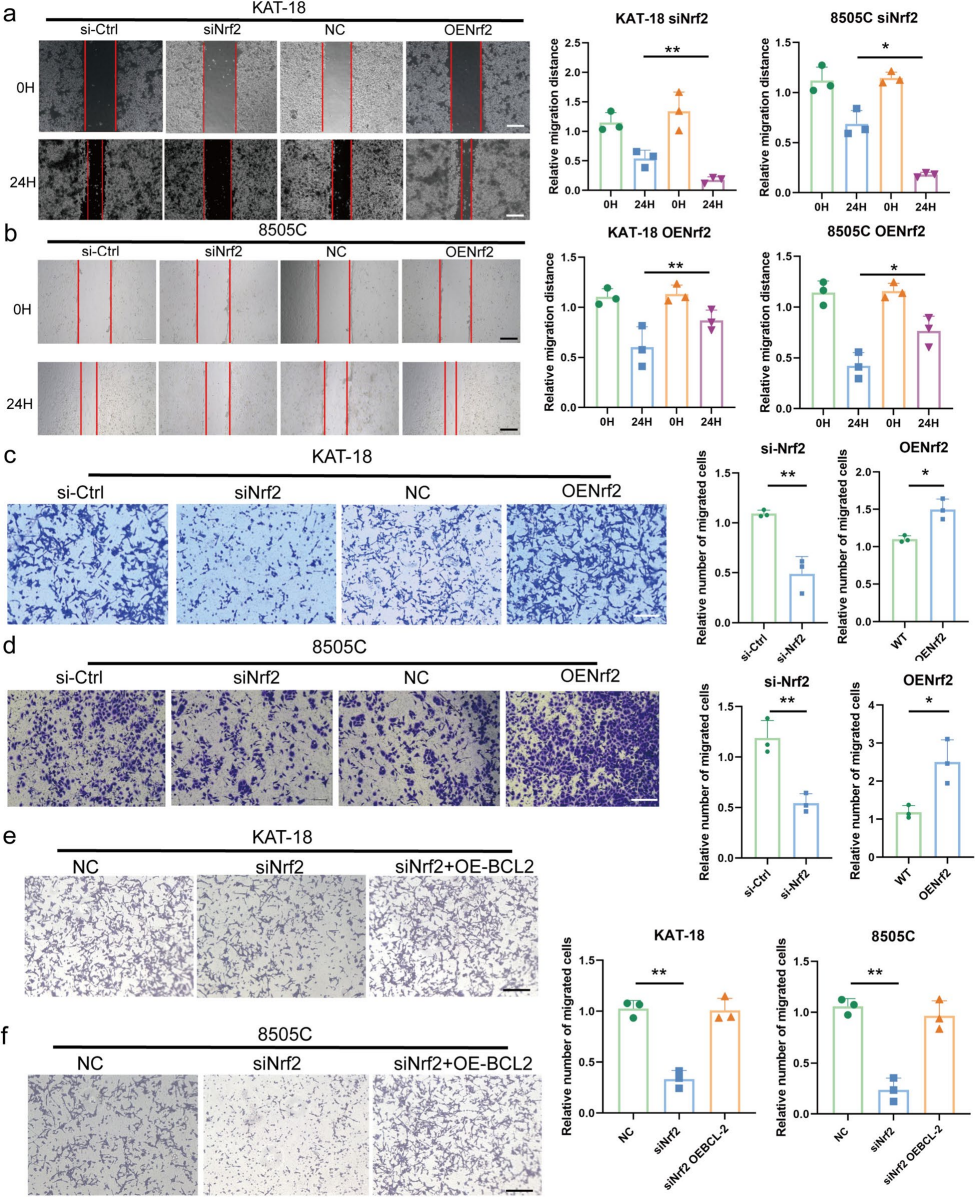
Silencing Nrf2 suppresses ATC growth and metastasis *in vitro.* **a**-**d** Wound healing (**a**-**b**) and transwell (**c**-**d**) assays evaluating the migration of ATC cells after Nrf2 knockdown or overexpression. **e**-**f** Rescue experiments showing that BCL-2 overexpression in siNrf2 cells partially restored migratory and invasive abilities in 8505 C and KAT-18 cells. The values are the means ± standard error of at least three separate studies. Compared with the corresponding control groups, * P<0.05, ** *P*<0.01

To assess the functional consequence of Nrf2-mediated anoikis resistance on metastasis, we evaluated cell migration. Nrf2 knockdown inhibited, while its overexpression enhanced, the migratory capacity of ATC cells in wound healing and transwell assays (Fig. 5a-d). Crucially, overexpression of BCL-2 in Nrf2-silenced cells partially restored their migratory ability (Fig. 5e-f), indicating that BCL-2 is a key functional mediator downstream of Nrf2. We next evaluated the therapeutic potential of Nrf2 inhibition. The Nrf2 inhibitor Brusatol suppressed ATC cell viability in a dose-dependent manner (Fig. S1h-i). Another inhibitor, ML385, similarly impaired proliferation and invasion (Fig. S1j-o), confirming that pharmacological blockade of Nrf2 compromises ATC cell survival.

### Pharmacological inhibition of Nrf2 suppresses ATC growth and metastasis *in vivo.*

**Fig. 6:**
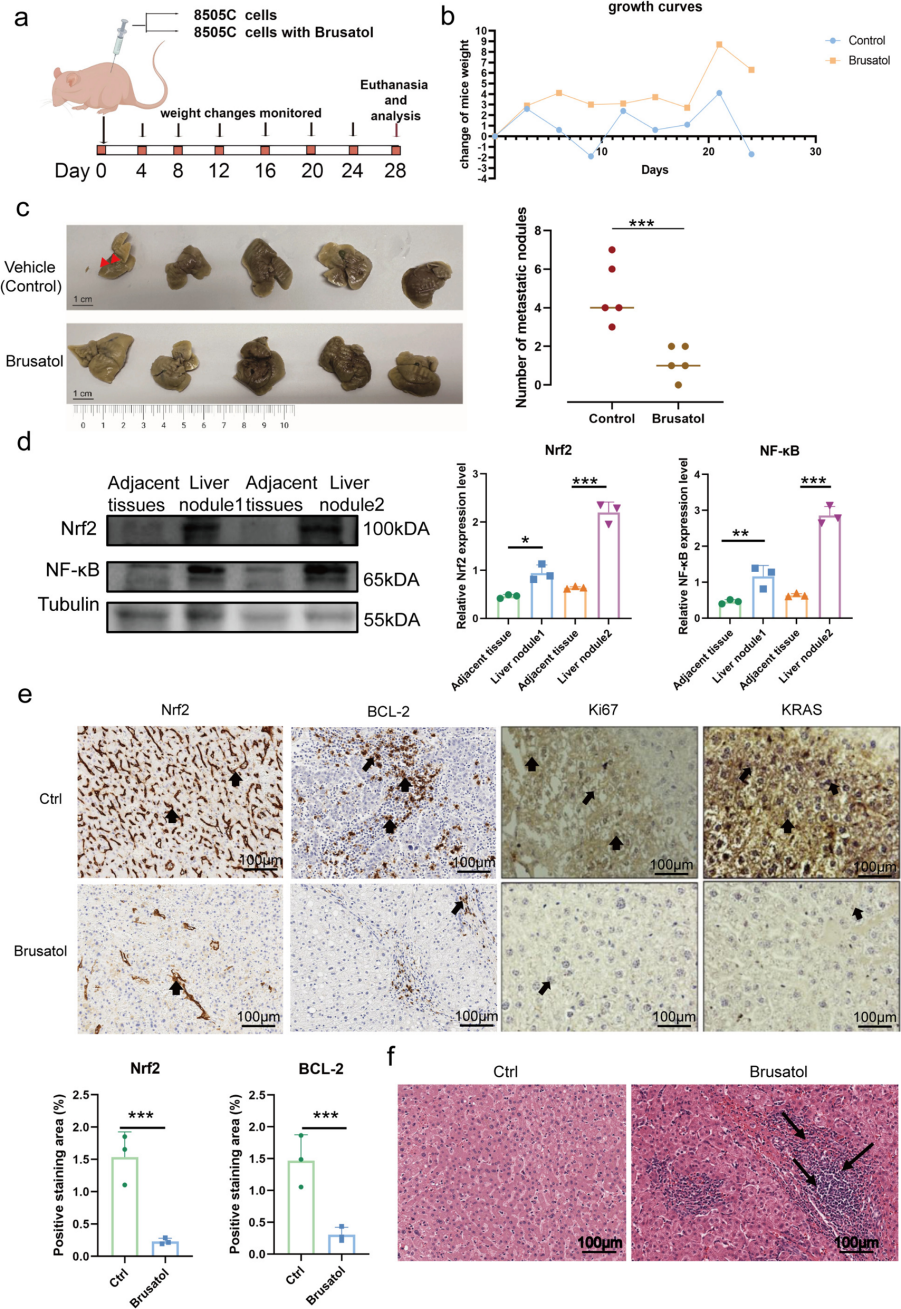
Pharmacological inhibition of Nrf2 suppresses tumor growth and liver metastasis of ATC *in vivo* (**a**) Schematic illustration of the *in vivo* experiment design: nude mice were injected with ATC cells via the tail vein and randomly assigned to vehicle or Brusatol treatment groups. (**b**) Body weight growth curve of nude mice in control and Brusatol-treated groups. Body weight was measured every 4 days. (**c**) Representative livers harvested from mice in the two groups; red arrows indicate metastatic nodules. (**d**) WB analysis of Nrf2 and NF-κB protein levels in liver metastatic nodules compared with matched adjacent non-tumor liver tissues, with densitometric quantification shown in Nrf2 and NF-κB. (**e**) Representative IHC staining of Nrf2, BCL-2, Ki67, and KRAS in liver metastatic nodules from nude mice treated with vehicle control or Brusatol. The control group refers to tumor-bearing mice receiving vehicle treatment. Arrows indicate positively stained regions. Scale bar, 100 µm. (**f**) H&E staining of liver metastatic tumors. The Brusatol group shows poor differentiation and enlarged necrotic areas (black arrows), compared to compact tumor structure in the control group. Scale Bar, 100 µm.The values are the means ± standard error of at least three separate studies. Compared with the corresponding control groups, * *P*<0.05, ** *P*<0.01, ***p < 0.001

We therefore selected Brusatol for *in vivo* validation, as our previous research had already demonstrated its efficacy in suppressing tumor growth in mouse xenograft models [30]. A nude mouse liver metastasis model was established by tail vein injection of 8505C cells with or without Brusatol (Fig. 6a). Body weight was monitored every four days. After 28 days, Brusatol-treated mice showed markedly fewer liver metastatic nodules, confirming that Nrf2 inhibition effectively suppresses ATC metastasis *in vivo* (Fig. 6b-c). Western blot analysis of metastatic nodules revealed elevated levels of Nrf2 and NF-κB p65 compared to adjacent normal tissue (Fig. 6d). Vehicle-treated tumor-bearing mice served as the control group, while Brusatol-treated mice showed markedly reduced Nrf2, BCL-2, Ki67, and KRAS expression in metastatic liver nodules [15, 31] (Fig. 6e). H&E staining further showed that Brusatol-treated tumors exhibited poor differentiation and extensive necrosis (Fig. 6f). These data collectively demonstrate that Nrf2 inhibition effectively suppresses ATC metastasis *in vivo*.

## Discussion

Genetic mutations and environmental factors contribute to thyroid cancer development [32], with ATC representing its most lethal form due to frequent distant metastasis at diagnosis[33–35]. This aggressive spread is facilitated by anoikis resistance, a capability for detached survival that underpins metastatic dissemination [36–39]. While mechanisms such as miRNA- and circRNA-mediated regulation of autophagy have been linked to anoikis resistance in other cancers like prostate carcinoma [40, 41], the pathways governing this process in ATC remain poorly defined. Diverging from these previously described mechanisms, our study identifies the transcription factor Nrf2 as a central regulator of anoikis resistance in ATC through a mechanism involving its nuclear translocation and transcriptional activation of specific target genes.

Nrf2 is well-established as a master regulator of the cellular antioxidant response, conferring protection by binding to AREs and transactivating cytoprotective genes [42]. This pathway is often co-opted in cancer, promoting aggressiveness and therapy resistance across various malignancies [43–46], including ATC [47]. For instance, Nrf2 activation driven by KEAP1 mutations accelerates lung cancer progression [48] while its inhibition can sensitize colorectal cancer cells to oxaliplatin-induced ferroptosis [49]. Consistent with its role as an oncogenic factor, we confirmed that elevated Nrf2 expression correlates with advanced tumor stage and poor survival in ATC. Building on this, our findings that Nrf2 is crucial for anchorage-independent survival and metastasis position it as a key therapeutic target.

Our functional data demonstrate that Nrf2 is not merely a correlative marker but a central executor of anoikis resistance in ATC. It fosters a pro-survival microenvironment under detachment stress, as evidenced by enhanced spheroid formation and a time-dependent induction that shifts the apoptotic balance via the BCL-2/BIM axis. Interestingly, this activation occurs independently of classical oxidative stress, relying instead on anoikis-induced KEAP1 ubiquitination and degradation to disrupt the Nrf2-KEAP1 complex and facilitate nuclear translocation.

Rapid BCL-2 induction confers early anti-apoptotic protection, while gradual SLC7A11 activation sustains redox balance. Combined functional and reporter analyses, together with prior ChIP data [27–29, 50], identify both as direct, non-redundant Nrf2 targets driving anoikis resistance.

Brusatol and ML385 effectively suppressed ATC cell viability and invasion. Brusatol treatment reduced liver metastasis and downregulated BCL-2 and Ki67, validating the functional impact of this axis. Concurrent KRAS suppression indicates disruption of a KRAS–Nrf2–BCL-2 loop, while the parallel decline of Nrf2 and NF-κB suggests pathway crosstalk in the metastatic niche [51].

This study elucidates the mechanism underlying Nrf2-driven tumor progression in ATC [15], identifying BCL-2 and SLC7A11 as key transcriptional mediators of anoikis resistance. Beyond detachment stress, Nrf2 broadly sustains ATC cell survival through anti-apoptotic and metabolic regulation. These findings establish Nrf2–ARE signaling as a central determinant of anoikis resistance and a promising therapeutic target in thyroid cancer.

Several limitations should be acknowledged. Although our data support direct transcriptional control, future ChIP analyses are required to confirm Nrf2 binding to the *BCL-2* and *SLC7A11* promoters. The lack of an orthotopic model and limited clinical samples also constrain the interpretation of metastatic dynamics. Moreover, the mechanisms underlying KEAP1 degradation during anoikis remain to be defined.

Overall, we establish Nrf2 as a central regulator of *BCL-2* and *SLC7A11* that confers anoikis resistance and promotes ATC metastasis, highlighting its potential as both a prognostic marker and therapeutic target.

## Materials and methods

### Cell culture

ATC cell lines KAT-18 and 8505C (previously obtained from ATCC [15]), PTC cell line BCPAP (DSMZ, Cat# ACC-273), and normal thyroid follicular epithelial cells Nthy-ori 3-1 (ECACC, Cat# 90011609) were cultured in Dulbecco’s Modified Eagle Medium (DMEM; Gibco, Life Technologies) supplemented with 10% heat-inactivated fetal bovine serum (FBS; Gibco, Life Technologies). All cell lines were maintained as monolayers at 37 °C in a humidified incubator with 5% CO₂ and used within 20 passages.

### Clinical samples

Seven pairs of paired ATC tissues and nearby non-cancer tissues were purchased from the third-affiliated Hospital of Harbin Medical University. The expression of Nrf2 protein in ATC and adjacent non-cancer tissues was detected by immunohistochemistry (IHC) after the tissues were formalin-fixed and paraffin-embedded. ATC tissues and their matched neighboring non-cancer counterparts were examined for Nrf2 expression. Informed permission was obtained by each participating patient, and the third affiliated Hospital of Harbin Medical University 's Ethics Committee approved the procedures (XJS2022-39).

### Bioinformatics analysis

Gene Expression Omnibus (https://www.ncbi.nlm.nih.gov/) provided the GSE33630 dataset [20]. The Seurat package in R version 4.0.4 was used to analyze bulk RNA-seq data, which included normalization, dimensionality reduction, and batch correction.

### Transfection assays

Small interfering RNAs (siRNAs) targeting Nrf2 (siNrf2#1, siNrf2#2, siNrf2#3) were obtained from Shanghai Bioscience Biotechnology Co., Ltd. Transfections were carried out using Lipofectamine 3000 (Invitrogen, USA) in serum- and antibiotic-free medium for 4 h, followed by replacement with complete medium for 18 h. Cells were then harvested for downstream analyses. The siRNA sequences are provided in Supplementary Table 1.

Plasmids for Nrf2 overexpression and mutants lacking NLS2 (pCMV-Nrf2-MUT1) or NLS3 (pCMV-Nrf2-MUT2) were obtained from Shanghai Bioscience Biotechnology Co., Ltd. Promoter-reporter plasmids containing the promoter regions of Nrf2, BCL-2, SLC7A11, and BIM, along with a Renilla luciferase control vector, were purchased from Beijing Tsingke Biotechnology Co., Ltd. Transfections were performed using Lipofectamine 3000 according to the manufacturer’s instructions. Cells were seeded in 6-well plates (60–70% confluence), transfected with 2–3 μg plasmid DNA per well, and harvested 24–48 h post-transfection for subsequent assays.

### Anoikis assay

1 × 10⁶ cells were suspended in SPL3D™ Cell Floater culture plates (SPL LIFE SCIENCES, Korea) and cultivated for 24 hours in anchor-age-independent conditions to create the anoikis model. Where indicated, Tetrathiomolybdate (TM, 1 μg/mL) was given to the suspension media to pharmacologically induce anoikis. After 24 hours, suspended cells were collected and put through further tests.

### Western Blot (WB)

Proteins were extracted from cultured cells or tumor tissues using RIPA lysis buffer supplemented with protease and phosphatase inhibitors and quantified with a Pierce™ BCA Protein Assay Kit (23225; Thermo Fisher Scientific, Waltham, MA, USA). Equal amounts of protein (30 μg per sample) were separated on 4%–12% Bis–Tris NuPAGE gels (EC6026BOX; Invitrogen™) and transferred onto PVDF membranes. After blocking with 5% non-fat milk, membranes were incubated overnight at 4 °C with primary antibodies against Nrf2 (ABclonal, A0674, 1:1000), KEAP1 (CST, 8047, 1:1000), GSK3β (CST, 9315, 1:1000), BCL-2 (CST, 15071, 1:1000), SLC7A11 (CST, 12691, 1:1000), BIM (CST, 2933, 1:1000), NF-κB p65 (CST, 8242, 1:1000), Cleaved Caspase-3 (CST, 9664, 1:1000), GAPDH (Santa Cruz, sc-47724, 1:2000), β-actin (CST, 4970, 1:2000), Lamin B1 (CST, 13435, 1:2000), and Histone H3 (CST, 4499, 1:1000). After washing, membranes were incubated with HRP-conjugated secondary antibodies (CST, 1:2000) for 1 h at room temperature. Protein bands were visualized using an Alpha Innotech chemiluminescence imaging system (ProteinSimple) with enhanced chemiluminescent substrate (ECL; Bio-Rad). Band intensity was quantified using ImageJ (NIH, USA). GAPDH and Lamin B1 or Histone H3 served as cytoplasmic and nuclear loading controls, respectively.

### Nuclear protein extraction

Nuclear and cytoplasmic fractions were isolated using the NE-PER™ Nuclear and Cytoplasmic Extraction Kit (Thermo Fisher Scientific) according to the manufacturer’s protocol. Briefly, cells were washed with cold PBS, lysed with Cytoplasmic Extraction Reagents I and II, and centrifuged to obtain the cytoplasmic fraction. The remaining pellets were extracted using Nuclear Extraction Reagent to obtain nuclear proteins. Protein concentrations were determined using the Pierce™ BCA Protein Assay Kit.

### Supershift Electrophoretic Mobility Shift Assay (Supershift EMSA)

An EMSA was performed using a Gel Shift Assay Kit (Beyotime, Shanghai, China) following the manufacturer’s protocol. Biotin-labeled antioxidant response element (ARE) oligonucleotides were synthesized by Beyotime. Nuclear extracts were obtained from 8505C cells cultured under suspension (24 h), or TM-treated conditions. For supershift assays, nuclear extracts were preincubated with anti-Nrf2 antibody (CST, 12721, 1:100) for 30 min on ice prior to probe addition. The DNA–protein complexes were resolved on 6% native polyacrylamide gels, transferred onto nylon membranes, and UV-crosslinked. Complexes were detected with streptavidin–HRP and visualized using the ChemiDoc™ MP Imaging System (Bio-Rad).

### Cell Proliferation assay

Cell viability was assessed using an MTT Cell Proliferation Kit (Sigma-Aldrich, St. Louis, MO, USA) following the manufacturer’s instructions. KAT-18 and 8505C cells were seeded in 96-well plates at a density of 3 × 10^3^ cells per well in 100 μL of complete medium, with or without Tetrathiomolybdate (TM, 1 μg/mL) treatment. After 24, 48, and 72 h of incubation, 10 μL of MTT solution (5 mg/mL) was added to each well and incubated for 4 h at 37 °C. The formazan crystals were dissolved in 100 μL of DMSO, and absorbance was measured at 570 nm using a BioTek microplate reader. Relative cell viability was expressed as the ratio of optical density (OD) values normalized to the control group.

### Migration assays

About 1 × 10^4^ cells were suspended in 200 μL of serum-free DMEM and seeded into the upper chambers of transwell inserts (8 μm pore, Millipore). 800 μL of complete medium was put into the lower chambers. Following 48 hours, migrating cells on the underside of the membrane were preserved with 4% paraformaldehyde and stained with 0.1% crystal violet (Sigma-Aldrich, St. Louis, MO, USA). The cells beneath the chamber surface were photographed, and the cells were counted in three random fields.

### Wound healing assay

Wound healing assays were performed in 6-well plates (Corning, USA) to evaluate cell migration. Cells from each treatment group were seeded and cultured until approximately 90% confluence. A sterile 200 μL pipette tip was used to create a linear scratch in the monolayer, followed by three gentle PBS washes to remove detached cells. The remaining cells were incubated in serum-free medium for 24 h. Images of the wound area were captured at 0 h and 24 h using an inverted microscope (Leica DMi8, Germany). Wound closure was quantified using ImageJ software (NIH, Bethesda, MD, USA) by calculating the percentage of wound area reduction relative to the initial width.

### IHC staining

Tumor tissues were fixed in 10% neutral-buffered formalin for 24 h, transferred to 70% ethanol, and paraffin-embedded. Sections (4 μm) were deparaffinized in xylene, rehydrated through graded ethanol, and subjected to antigen retrieval in 10 mM sodium citrate buffer (pH 6.0) at 95 °C for 20 min. Endogenous peroxidase activity was quenched with 3% H₂O₂. After blocking with 5% BSA, slides were incubated overnight at 4 °C with anti-Nrf2 antibody (ABclonal, A0674, 1:200), followed by HRP-conjugated secondary antibody (Abcam, ab6721, 1:500) for 1 h at room temperature. Signal was developed using DAB (Vector Laboratories, SK-4100) and counterstained with hematoxylin. Images were acquired using a Nikon ECLIPSE Ni microscope. Quantification was performed in five random 20× fields per sample using ImageJ with color deconvolution, and the percentage of positive cells was averaged per case.

### TUNEL assay

Apoptotic cells were detected using a TUNEL Assay Kit (Promega, Madison, WI, USA) following the manufacturer’s protocol. KAT-18 and 8505C cells were seeded on sterilized coverslips in 24-well plates and cultured under different treatments for 48 h. Cells were fixed with 4% paraformaldehyde for 30 min, permeabilized with 0.3% Triton X-100 for 5 min and incubated with the TUNEL reaction mixture for 60 min in the dark. Nuclei were counterstained with DAPI and mounted using antifade medium. Fluorescence images were acquired using a Leica DMi8 fluorescence microscope, and the proportion of TUNEL-positive cells was quantified using ImageJ software.

### Immunofluorescence assay

Following 24 h of TM treatment, KAT-18 and 8505C cells were fixed with 4% paraformaldehyde for 15 min at room temperature, permeabilized with 0.3% Triton X-100 for 10 min, and blocked with 5% BSA for 1 h. Cells were incubated overnight at 4 °C with primary antibodies against Nrf2 (ABclonal, A0674, 1:100), SLC7A11 (ABclonal, A13685, 1:100), or BCL-2 (ABclonal, A20777, 1:100), followed by fluorophore-conjugated secondary antibodies (Jackson ImmunoResearch, 1:500) for 1 h at room temperature in the dark. Nuclei were counterstained with DAPI and mounted using SlowFade™ Gold Antifade Mountant (Thermo Fisher Scientific, Waltham, MA, USA). Images were captured using a confocal laser scanning microscope (Carl Zeiss, Germany).

### Dual-Luciferase reporter assay

Promoter fragments of human BCL-2 (−2000 to +100 bp), BIM (−1500 to +50 bp), and SLC7A11 were synthesized and cloned into the pGL3-Basic luciferase vector (Promega, Madison, WI, USA) by Beijing Tsingke Biotech Co., Ltd. All constructions were verified by Sanger sequencing. KAT-18 and 8505C cells were seeded in 24-well plates (1 × 10^5^ cells/well) and transfected with 400 ng of firefly luciferase reporter plasmid and 40 ng of Renilla control plasmid using Lipofectamine 3000 (Invitrogen, USA). After 24–48 h, luciferase activity was measured using the Dual-Luciferase® Reporter Assay System (Promega). Firefly luciferase activity was normalized to Renilla luciferase activity to control for transfection efficiency.

### Immunoprecipitation (Co-IP)

KAT-18 cells (1 × 10⁷) were cultured for 24 h in SPL3D™ Cell Floater plates (SPL Life Sciences, Korea) under anchorage-independent conditions and lysed with ice-cold IP lysis buffer containing protease inhibitors (Beyotime, Shanghai, China). Cell lysates were clarified by centrifugation at 12,000 × g for 10 min at 4 °C. The supernatants were incubated overnight at 4 °C with anti-KEAP1 (CST, 8047), anti-Ubiquitin (ABclonal, A23483), or normal IgG (ABclonal, A19711). Protein A/G magnetic beads (Thermo Fisher Scientific, Waltham, MA, USA) were then added and incubated for 4 h, followed by washing with IP lysis buffer. Bound proteins were eluted with 2× SDS loading buffer and denatured at 95 °C for 10 min. Immunoprecipitates were analyzed by western blotting.

### Quantitative Real-Time PCR (qRT-PCR)

Total RNA was isolated from KAT-18 and 8505C cells using Trizol (Invitrogen) and reverse transcription using PrimeScript™ RT Kit (Takara). qRT-PCR was conducted with SYBR Green Master Mix (Takara) on an ABI 7500 machine (Applied Biosystems). GAPDH was internal control. Relative gene expression was calculated using the 2^−ΔΔCt technique. Supplementary Table 2 contains primer sequences.

### *In Viv*o xenograft assay

Female BALB/c nude mice (4–6 weeks old) were purchased from the Laboratory Animal Service Center of The Chinese University of Hong Kong. Mice were maintained under a 12-h light/dark cycle with free access to food and water. A total of 10 mice were randomly divided into two groups (*n* = 5 per group): control and Brusatol treatment. Each mouse received a subcutaneous injection of 1 × 10⁶ 8505C cells. Starting on the day of inoculation, mice were administered Brusatol (2 mg/kg, i.p.) or vehicle (0.9% saline, i.p.) every other day. Body weight was recorded every three days. After 28 days, mice were euthanized, and liver tissues were collected for western blot and immunohistochemistry (IHC) analyses of Nrf2 and related markers. All animal experiments were approved by the Animal Ethics Committee of The Chinese University of Hong Kong (Ref. No. 23–1317 in DH/HT&A/8/2/1 Pt.57).

### Statistical analysis

Data were analyzed using SPSS 24.0 (IBM) and GraphPad Prism 8.0. Data are presented as the mean ± standard deviation (SD) from at least three independent experiments. Statistical significance between two groups was determined by the student’s t-test. Comparisons among multiple groups were performed using one-way analysis of variance (ANOVA) followed by Tukey's post hoc test. *P* < 0.05 was considered statistically significant.

## Supplementary Information

Supplementary Material 1.

## Data Availability

The datasets generated and/or analyzed during the current study are available from the corresponding author upon reasonable request.
