# Supplementary material for: The role of Nrf2 in anoikis resistance and metastasis in anaplastic thyroid carcinoma
Source: Mol Biomed. 2025 Nov 10;6:103. doi: 10.1186/s43556-025-00355-7 (PMC12602778; doi:10.1186/s43556-025-00355-7)
Supplement: Supplementary file 1 — Supplementary Material 1. [file 43556_2025_355_MOESM1_ESM.pdf]

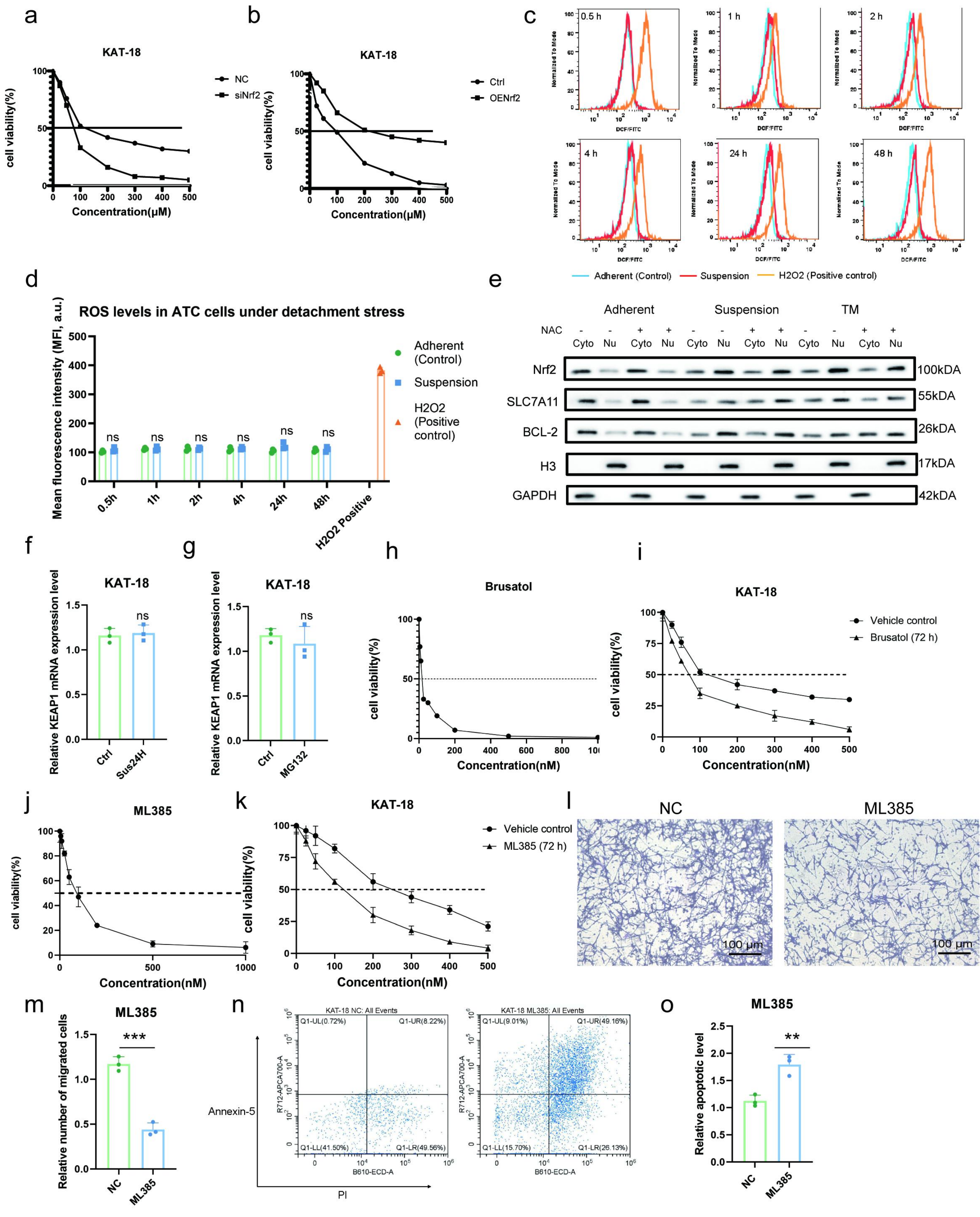

a
KAT-18
NC
siNrf2
cell viability(%)
Concentration(μM)
b
KAT-18
Ctrl
OENrf2
cell viability(%)
Concentration(μM)
c
0.5 h
1 h
2 h
4 h
24 h
48 h
Normalized To Mode
DCF/FITC
Adherent (Control)
Suspension
H2O2 (Positive control)
d
ROS levels in ATC cells under detachment stress
Mean fluorescence intensity (MFI, a.u.)
Adherent (Control)
Suspension
H2O2 (Positive control)
ns
0.5h
1h
2h
4h
24h
48h
H2O2 Positive
e
Adherent
Suspension
TM
NAC
Cyto
Nu
Nrf2
SLC7A11
BCL-2
H3
GAPDH
100kDA
55kDA
26kDA
17kDA
42kDA
f
KAT-18
Relative KEAP1 mRNA expression level
ns
Ctrl
Sus24H
g
KAT-18
Relative KEAP1 mRNA expression level
ns
Ctrl
MG132
h
Brusatol
cell viability(%)
Concentration(nM)
i
KAT-18
Vehicle control
Brusatol (72 h)
cell viability(%)
Concentration(nM)
j
ML385
cell viability(%)
Concentration(nM)
k
KAT-18
Vehicle control
ML385 (72 h)
cell viability(%)
Concentration(nM)
l
NC
ML385
100 μm
100 μm
m
ML385
Relative number of migrated cells
***
NC
ML385
n
KAT-18 NC: All Events
KAT-18 ML385: All Events
Q1-UL(0.72%)
Q1-UR(8.22%)
Q1-LL(41.50%)
Q1-LR(49.56%)
Q1-UL(9.01%)
Q1-UR(49.16%)
Q1-LL(15.70%)
Q1-LR(26.13%)
R712-APCA700-A
B610-ECD-A
Annexin-5
PI
o
ML385
Relative apoptotic level
**
NC
ML385

Supplementary Figure 1. Silencing Nrf2 inhibits ATC cell proliferation.
(a – b) Cell viability of ATC cells under TM-induced anoikis conditions. (a) Knockdown of Nrf2 reduced viability of KAT-18 cells. (b) Overexpression of Nrf2 enhanced viability and increased the IC under detachment stress. Cell viability was measured by MTT assay after 24 h. **(c – d)**Flow cytometric analysis of intracellular ROS in ATC cells cultured under suspension (0.5 – 48 h) or adherent conditions. H2O2 treatment served as a positive control. **(e)**Nuclear and cytoplasmic fractionation of Nrf2, BCL-2, and SLC7A11 in ATC cells cultured under three conditions (adherent, suspension, TM + suspension) with or without NAC supplementation. NAC treatment did not significantly affect protein distribution, suggesting that the effect is independent of ROS. **(f – g)** Expression of KEAP1 mRNA in KAT-18 cells after 24 h suspension (f) and after MG132 treatment (g), detected by RT-qPCR. **(h – i)** Effects of Brusatol on ATC cell proliferation. (h) Cell viability was determined by MTT assay. (i) IC50 of Brusatol was approximately **180 nM**. **(j – k)** Effects of ML385 on ATC cell proliferation .Cell viability and IC50 were determined by MTT assay under the same conditions as in (h – i).The estimated IC50 of ML385 was approximately **220 nM.(l – m)**Transwell migration assay comparing ML385-treated vs. control ATC cells. **(n – o)**Flow cytometric analysis of the effect of ML385 on ATC cells. The values are the means ± standard error of at least three separate studies. Compared with the corresponding control group,$^{**}p < 0.01$, $^{***}p < 0.001$, ns: not significant.

Supplementary table1 Nrf2 siRNA sequences

Nrf2-siRNA-1	CCGGCATTTCACTAAACACAA
Nrf2-siRNA-2	AGTTTGGGAGGAGCTATTATC
Nrf2-siRNA-3	atccattcctgagttacagtg

Supplementary table2 Primer sequences

Target mRNA	Forward primer (5-3')	Reverse primer (5'-3‘)
Nrf2	TTGGCCCAAGAAGCTTGA	CTTGCCTCTCCTGCGTAT
KEAP1	CTTGCCTCTCCTGCGTAT	TGAAGAACTCCTCCTGC
SLC7A11	TCCCATTTCTGTTGCCATGTC	GCCTATCAAGGAACCAATCGG
BCL-2	ATCGCCCTGTGGATGACTGAGT	GCCAGGAGAAATCAAACAGAGGC
BIM	GCCCCTACCTCCCTACAGAC	CCTTATGGAAGCCATTGCAC
GAPDH	CCATGTTCGTCATGGGTGTG	GGTGCTAAGCAGTTGGTGGTG
